# Editorial Note: Preclinical evaluation of 4-methylthiobutyl isothiocyanate on liver cancer and cancer stem cells with different p53 status

**DOI:** 10.1371/journal.pone.0344076

**Published:** 2026-03-06

**Authors:** The PLOS One Editors

Following the publication of this article [1], concerns were raised with results presented in Figs 3 and 6. Specifically,

- The Huh7 β-actin panels of Fig 6C appear similar to the Huh7 middle and bottom β-actin panels in Fig 2A of [2].
- The Hep3B β-actin panels of Fig 6C appear similar to the Hep3B bottom β-actin panels in Fig 2A of [2].

The corresponding author stated that in order to allow direct comparability of the respective target protein signals with the corresponding β-actin loading control, β-actin loading controls were shown more than once in cases where the same membrane was used for the detection of different target proteins. The corresponding author also stated that the original raw data for [1] are no longer available.

The *PLOS One* Editors issue this Editorial Note to provide readers with the above information.

## References

[1] Lamy E, Hertrampf A, Herz C, Schüler J, Erlacher M, Bertele D, et al. Preclinical evaluation of 4-methylthiobutyl isothiocyanate on liver cancer and cancer stem cells with different p53 status. PLoS One. 2013;8(8):e70846. doi: 10.1371/journal.pone.0070846 23936472 PMC3732292

[2] Lamy E, Herz C, Lutz-Bonengel S, Hertrampf A, Márton M-R, Mersch-Sundermann V. The MAPK pathway signals telomerase modulation in response to isothiocyanate-induced DNA damage of human liver cancer cells. PLoS One. 2013;8(1):e53240. doi: 10.1371/journal.pone.0053240 23382840 PMC3561400

